# Developing the Key Driver Diagram by Analyzing Home Central Line Caregiver Proficiency Factors

**DOI:** 10.1097/pq9.0000000000000638

**Published:** 2023-03-13

**Authors:** Chris I. Wong, Natalie Henrich, Constance M. Barysauskas, Margaret Conway, Marie D. Desrochers, Riley M. Mahan, Amy L. Billett

**Affiliations:** From the * Pediatric Hematology-Oncology, Boston Children’s Hospital, Boston, Mass.; † Pediatric Oncology, Dana-Farber Cancer Institute, Boston, Mass.; ‡ Ariadne Labs at Brigham and Women’s Hospital and the Harvard T.H. Chan School of Public Health, Boston, Mass.; § Department of Biostatistics and Computational Biology, Dana-Farber Cancer Institute, Boston, Mass.

## Abstract

Caregivers of pediatric oncology and stem cell transplant patients often care for central lines (CLs) at home. Methods to achieve caregiver CL care proficiency, and interventions designed with caregiver input are lacking.

**Methods:**

Caregivers of pediatric oncology and stem cell transplant patients patients with an external CL or removed within 2 weeks were eligible for a survey assessing knowledge, the value of training strategies, and comfort. We mapped responses (n = 79) and acceptability/challenges of introducing a pilot caregiver CL teach-back clinic program onto the capability, opportunity, motivation behavioral (COM-B) model of change to identify drivers of caregiver CL care proficiency. A working group, including caregivers, refined and approved a final driver diagram.

**Results:**

*Survey*: Ninety-four percent of caregivers answered knowledge questions correctly (capability); 95% considered hands-on training helpful (opportunity); 53% were not very comfortable with CL care (motivation). *Teach-back*: Seventy-nine percent of caregivers were interested in a teach-back as additional training; 38% participated (opportunity); 20% refused participation due to being overwhelmed/not having time (motivation). Thirty-three percent of participants had a CL proficiency assessment (capability). Drivers of home caregiver CL care proficiency included: support for the caregiver’s physical capability to perform CL care; enabling the CL care nurse trainer role; facilitating and increasing training opportunities, and engaging caregivers early and continuously to motivate proficiency development appropriately.

**Conclusions:**

An approach centered on caregivers as main stakeholders can identify drivers to co-design an intervention for improved home CL care delivery. A standardized process to train and evaluate caregivers with multiple hands-on opportunities might be beneficial.

## INTRODUCTION

Caregivers (eg, parents and guardians) of pediatric oncology and stem cell transplant (POSCT) patients are often responsible for daily home care of central lines.^1^ Adherence to an evidence-based care bundle prevents central line-associated bloodstream infections (CLABSI).^2–4^ These infections cause prolonged hospital stays costing more than $35,000, 15% require intensive care, and 40% require CL surgical removal.^5^ Preventing ambulatory CLABSI may necessitate bundle adherence by patients and caregivers at home, in addition to healthcare staff.^1–8^ While caregivers can feel unprepared with medical devices at home, interventions are not often designed with their input or focus on optimizing caregiver proficiency.^1,2,9–19^ We sought to add to the limited literature of interventions co-designed with caregivers.

Despite years of maximizing healthcare staff bundle adherence in the ambulatory setting, our center’s POSCT ambulatory CLABSI rate remained unchanged (0.3 per 1000 CL days). In addition, we did not track caregiver CL care proficiency or had efforts to optimize this.

Before embarking on an improvement initiative, we performed a current state analysis (CSA) to assess home caregivers’ perceptions around our program’s CL care training to understand drivers of caregiver CL care proficiency. The goal was to obtain caregivers’ input through the CSA before a pilot QI intervention. The first output was the development of a key driver diagram (KDD),^20,21^ with a specific, measurable, attainable, relevant, time-bound (SMART) aim to achieve >90% of caregivers independent and comfortable with CL care after 1 year. This initiative was part of a larger ambulatory CLABSI prevention initiative.

## METHODS

### Setting

We carried out the study in a tertiary care, university-affiliated POSCT center from May 2015 to May 2016. We created a sub-group from an existing multidisciplinary CLABSI prevention committee. The sub-group had physician representation with POSCT, CLABSI prevention, and QI expertise; ambulatory and inpatient nurse champions; and a population health manager. The larger committee also included 2 members of our pediatric patient and family advisory council (PPFAC), infection control specialists, and other providers. PPFAC members provided feedback during meetings. The committee met monthly, and a sub-group met weekly. The sub-group brainstormed interventions to achieve caregiver independence and comfort with CL care, bringing concepts to the larger group. A logic model that visually depicted the chain of cause and effects leading to an outcome(s) and desired aim [improving ambulatory CLABSI rates (**See figure, Supplemental Digital content 1,** http://links.lww.com/PQ9/A463)] served as a guide.^22,23^ We conducted a CSA as the first step in the QI intervention design to understand drivers of home caregiver CL care proficiency.

### Current State of CL Care Caregiver Training

We adapted the Centers for Disease Control and Prevention National Healthcare Safety Network CL care maintenance care bundle for use by caregivers at home.^2,4,24^ This bundle was designed for healthcare staff use, not home caregivers. Before the pilot QI intervention, we performed caregiver CL training almost exclusively in the inpatient setting just before hospital discharge. Hospital nurses trained caregivers using instruction sheets and hands-on practice. There was no standardization on the timing, duration, extent, and methodology, but rather, they were determined by individual nurses based on a subjective evaluation of caregiver proficiency. No formal process to train the trainers or a checklist existed. Caregivers received no additional CL care training or evaluation after hospital training once the patient arrived home or returned to the clinic. Some patients had CL care reinforcement with nonaffiliated home health nurses, but our POSCT center did not coordinate this.

### Participants

We recruited caregivers of POSCT patients with an external CL for a survey. We also included caregivers of patients with a CL removed within the last 2 weeks. We assumed patients with recent CL removal had completed therapy and would have more time to complete a survey but still have reliable insight. We did not survey patients who cared for their own CL. Surveys were in English, but we included patients with an in-person interpreter present during routine visits. Patients were identified through an existing home-grown database to track CL days. We approached caregivers during routine clinic visits or hospital stays during downtime when active cancer care would not be interrupted (eg, during chemotherapy infusion). We approached 1 caregiver per patient, primarily responsible for the CL. We confirmed this role by asking the patient’s nurse before approaching, as nurses routinely reviewed this information with patients. Data collected included limited demographic information (age, gender, primary caregiver language) and clinical facts (disease group and external CL history). After survey completion, the manager collected the survey and, if still present, asked the caregiver about interest in piloting clinic teach-backs.

### Surveys

The sub-group initially developed a 10-question survey; then refined it with the assistance of a survey design expert. We designed the survey at a third-grade reading level. Three caregivers piloted the survey; none suggested changes. REDCap, an electronic data capture tool, served as the centralized location for data collection and management.

There were 5 knowledge questions about the CL maintenance care bundle^4,24^ and risks. We provided answers after completion.

A 4-point Likert scale [indispensable (cannot do without it), helpful, sort of helpful, and not helpful (with 2 additional options of “not sure” and “does not apply”)] evaluated the value of existing training strategies: hands-on training by program nurses, hands-on training by home health nurses, and CL care instruction sheets. Finally, an open-ended free-text question asked caregivers for additional home CL care training strategies.

A 4-point Likert scale [very comfortable, comfortable, sort of comfortable, and not comfortable (with a fifth option of “not sure”)] determined caregiver CL care comfort level.

### Pilot Teach-backs

Before the pilot QI intervention, no subsequent caregiver CL training existed after the initial training. We sought to pilot the acceptability and challenges of introducing a new training strategy, a teach-back, in the clinic. A teach-back is a technique frequently used to consolidate a patient’s understanding of medical care information or skills. Teach-backs are associated with improved health literacy and outcomes.^25,26^ During the pilot teach-backs, caregivers would return-demonstrate CL care in their child or a mannequin with an expert nurse (trainer). The population health manager scheduled teach-backs with any available nurse during an upcoming clinic visit. We aimed to have nurses assess caregiver CL care proficiency during the teach-back, document proficiency in the medical record, and collect additional feedback caregivers provided about CL care training. Proficiency evaluation and obtaining feedback were not standardized processes. We summarized feedback based on themes. We tracked the percentage of caregivers interested and participating in teach-backs.

### COM-B Model

We mapped survey results, teach-back participation, and feedback onto the capability, opportunity, motivation behavioral (COM-B) model to understand drivers of caregiver CL care proficiency. The COM-B model offers a framework to evaluate fundamental components of behavior change to design interventions.^27–29^ The 3 COM-B model components are further subdivided into different aspects. Capability refers to the (physical and psychological) ability to engage in a behavior. Opportunity refers to external factors (physical and social) that allow the execution of a behavior. Motivation refers to the internal processes (automatic and reflective) influencing behavior. COM-B assumes that capability and opportunity influence motivation. To change behavior, researchers need to change 1 or more COM-B components.^27–29^

We aimed to address each component through at least 1 question in the survey and/or teach-back (Table 1). If an evaluation could assess more than 1 component, we determined only 1 to be the primary component assessed. We mapped themes from surveys and teach-backs onto all components.

**Table 1.** Caregiver Central Line Proficiency Evaluation Strategies Mapped into the Capability, Opportunity, Motivation Behavioral Model of Change

Component	Aspect	Question of Interest	Evaluation Strategy
Capability	Psychological	Do caregivers have the knowledge to perform correct CL care?	Survey1. Knowledge2. Other helpful training strategies (open-ended)*
	Physical	Is the caregiver able to independently carry out CL care correctly?	Teach-back:1. CL care proficiency evaluation
Opportunity	Physical	Do caregivers have appropriate resources to train in CL care?	Survey 1. Value of Training Strategies 2. Other helpful training strategies (open-ended)
		Do caregivers have the right environment to train in CL care outside the hospital?	Teach-back: 1. Participation (of those interested) 2. Feedback*
	Social	Is there a social pressure (or desirability) to train in CL care?	Teach-back: Interest
Motivation	Automatic	How do caregivers feel about caring for a CL at home?	Survey: Comfort
		How do caregivers feel about a new CL care training opportunity?	Teach-back: 1. Refusal Reasons 2. Feedback
	Reflective	Do caregivers have the appropriate beliefs about consequences of incorrect CL care?	Survey: 2 knowledge questions 1. Is washing your hands important to prevent line infections? 2. How serious are central line infections?

*Secondary domain evaluated by strategy.

CL, central line.

### KDD Development and Co-Design

We developed the SMART aim from our goal to standardize caregiver CL training and proficiency evaluation. First, the sub-group used summarized survey and teach-back data to derive an initial KDD, which was then presented to the CLABSI prevention group with patient-family advocates. Next, we iterated and refined the wording of drivers using suggested changes from the group and together brainstormed additional potential change ideas. Ultimately, the group approved a final KDD. Input from PPFAC members was also actively sought during the co-design. For example, if changes proposed during meetings could affect patients and caregivers, we ensured PPFAC members could provide feedback before incorporating the change idea onto the driver diagram.

### Statistical Analysis

Descriptive statistics characterized the patient population, responses, and participation rates. The helpfulness of each strategy was dichotomized into helpful or not (indispensable and helpful versus all others) to better categorize usefulness and understand which strategies were indispensable. We calculated exact binomial confidence intervals (CI). We dichotomized the responses to comfort based on time with the CL (less than 3 months versus not) to assess whether differences existed based on experience; we used a chi-square test for statistical significance. We used SAS 9.4 (Cary, N.C.) for statistical analysis.

We undertook this project as QI and, as such, was not formally supervised by the program’s Institutional Review Board per their policies. Therefore, we did not obtain informed consent.

## RESULTS

### Participants

**Fig. 1.**
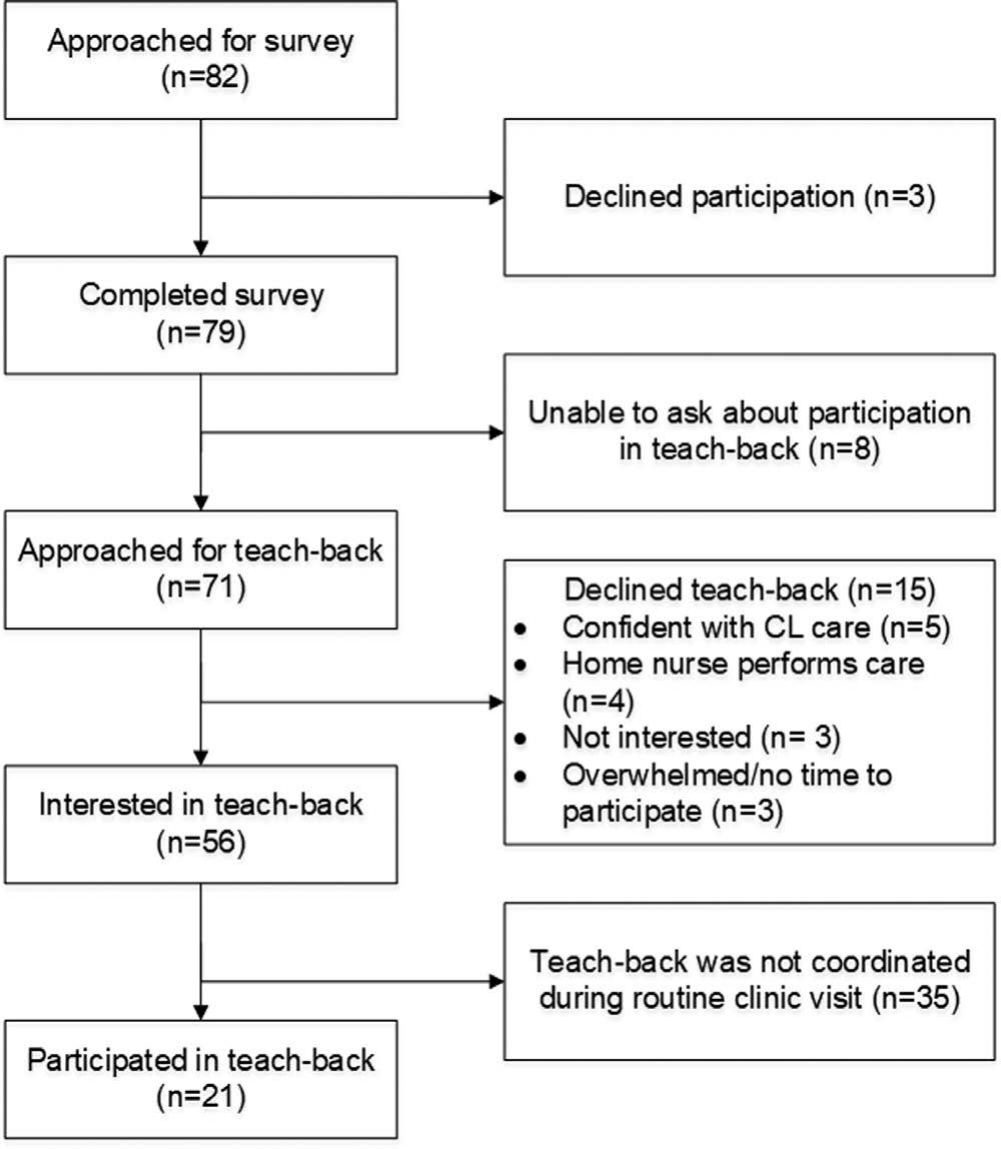
Consort diagram of caregiver recruitment and participation in surveys and pilot teach-backs.

Figure 1 demonstrates recruitment and participation. Of 82 caregivers approached, 79 (96%) completed the survey. The median age of patients was 5 [interquartile range (IQR) 2.3–11.9]. Forty-nine percent received a stem cell transplant. English was the primary language of 86% of caregivers. The median time with the external CL was 3 months (IQR 1.7–5.6); 68% of central lines were tunneled external catheters (Table 2).

**Table 2.** Survey Participant Characteristics

	**Total** (n = 79)
Patient demographics	
Age (years), median (IQR)	5 (2.3−11.9)
Female, n (%)	31 (39)
Disease group, n (%)	
Stem cell transplant	39 (49)
Solid tumor	24 (30)
Hematologic malignancy	11 (14)
Central nervous system tumor	5 (6)
Caregiver’s demographics	
Primary language, n (%)	
English	68 (86)
Spanish	4 (5)
Arabic	3 (4)
Other*	4 (5)
Interpreter preferred, n (%)	9 (11)
External central line characteristics	
Time from insertion to survey (months), median (IQR)	3 (1.7−5.6)
Current type, n (%)	
Tunneled external catheter	54 (68)
Peripherally inserted central catheter	25 (32)
Previous ambulatory CLABSI, n (%)	15 (19)

*Includes Bengali (n=1), Chinese Mandarin (n=1), Portuguese (n=2).

IQR, interquartile range; CLABSI, central line-associated bloodstream infection.

### Survey Responses

Table 3 displays survey responses. The COM-B Model component addressed is below, with aspects in parenthesis.

**Table 3.** Survey Responses Mapped onto the Capability, Opportunity, and Motivation Behavioral Model of Change

**Capability (psychological) questions**	**Correct Answers, n (%)**
Is washing your hands important to prevent line infections? (n=77)	77 (100)
When should central line dressings be changed? (n=77)	77 (100)
When must a mask be worn? (n=77)	74 (96)
When must central line caps be scrubbed with alcohol? (n=76)	75 (99)
How serious are central line infections? (n=77)	77 (100)
**Opportunity (physical): value of training strategies questions**	
Hands-on training: Program nurses (n=78)	
Indispensable, n (%)	41 (53)
Helpful, n (%)	33 (42)
Sort of helpful, n (%)	4 (5)
Not helpful, n (%)	0 (0)
Not sure, n (%)	0 (0)
Hands-on training: home health nurses (n = 61)	
Indispensable, n (%)	22 (36)
Helpful, n (%)	31 (51)
Sort of helpful, n (%)	6 (10)
Not helpful, n (%)	2 (3)
Not sure, n (%)	0 (0)
Instructions sheets (n =74)	
Indispensable, n (%)	14 (19)
Helpful, n (%)	32 (43)
Sort of helpful, n (%)	24 (33)
Not helpful, n (%)	3 (4)
NOT SURE, N (%)	1 (1)
**Motivation (automatic) questions**	
Caregiver comfort with CL care, n (%)	
Very comfortable	37 (47)
Comfortable	33 (42)
Sort of comfortable	8 (10)
Not comfortable	1 (1)
Not sure	0 (0)

*Capability* (psychological): Seventy-two of 77 (94%) caregivers answered all knowledge questions correctly.

*Opportunity* (physical): Fifty-nine of 79 caregivers (75%) received all 3 training strategies. Fifty-three percent valued hands-on training by program nurses as indispensable. Upon dichotomizing the 5 options into helpful or not helpful (indispensable and helpful versus all others), 95% identified hands-on training by program nurses as helpful (95% CI: 87–99%), 87% found home health nurses’ hands-on training helpful (95% CI: 76–94%), and 62% identified instruction sheets as helpful (95% CI: 50–73%).

Twenty caregivers commented on strategies helpful for learning CL care. Fifteen requested more opportunities for hands-on training and/or development of user-friendly quick reference tools to guide CL care (eg, a checklist). Other strategies included using CL care teaching videos (n = 2) and standardization of the training process (n = 2).

*Motivation* (automatic): Forty-seven percent of caregivers expressed the highest comfort level (very comfortable) with CL care. Of caregivers very comfortable with CL care, 46% had a child with a CL for at least 3 months versus 54% for less than 3 months. Comfort level responses did not differ significantly based on the time with CL (p=0.57). All caregivers (n=77) correctly answered the 2 survey questions exploring reflective motivation.

### Pilot Teach-backs Mapped onto the COM-B Model

#### Capability (physical)

Of caregivers participating in teach-backs, 7 (33%) had medical record documentation of CL care proficiency assessment. All caregivers assessed were independent with CL care.

#### Opportunity

We invited 71 out of 79 caregivers to a teach-back; 56 (79%) were interested (social). Twenty-1 out of 56 (38%) interested caregivers completed a teach-back (physical). Thirty-five caregivers could not complete the teach-back as we did not yet have a structured process to incorporate teach-backs as part of routine clinic visits during the pilot teach-back phase. As a result, we prioritized routine care without focusing on embedding teach-backs during the visit (eg, we did not embed downtime while waiting for laboratory results to perform a teach-back).

#### Motivation (automatic)

Reasons for not participating in teach-backs included: feeling confident with CL care (33%), having a home nurse (27%), not interested (20%), and feeling too overwhelmed and/or having no time to participate (20%). Three themes emerged from teach-back participant feedback: caregivers viewed teach-backs as a “test,” creating more anxiety; teach-backs were not efficiently incorporated into routine visits, consequently extending visits; and discrepancies existed on the timing, process, and content provided by trainers.

### KDD

**Fig. 2.**
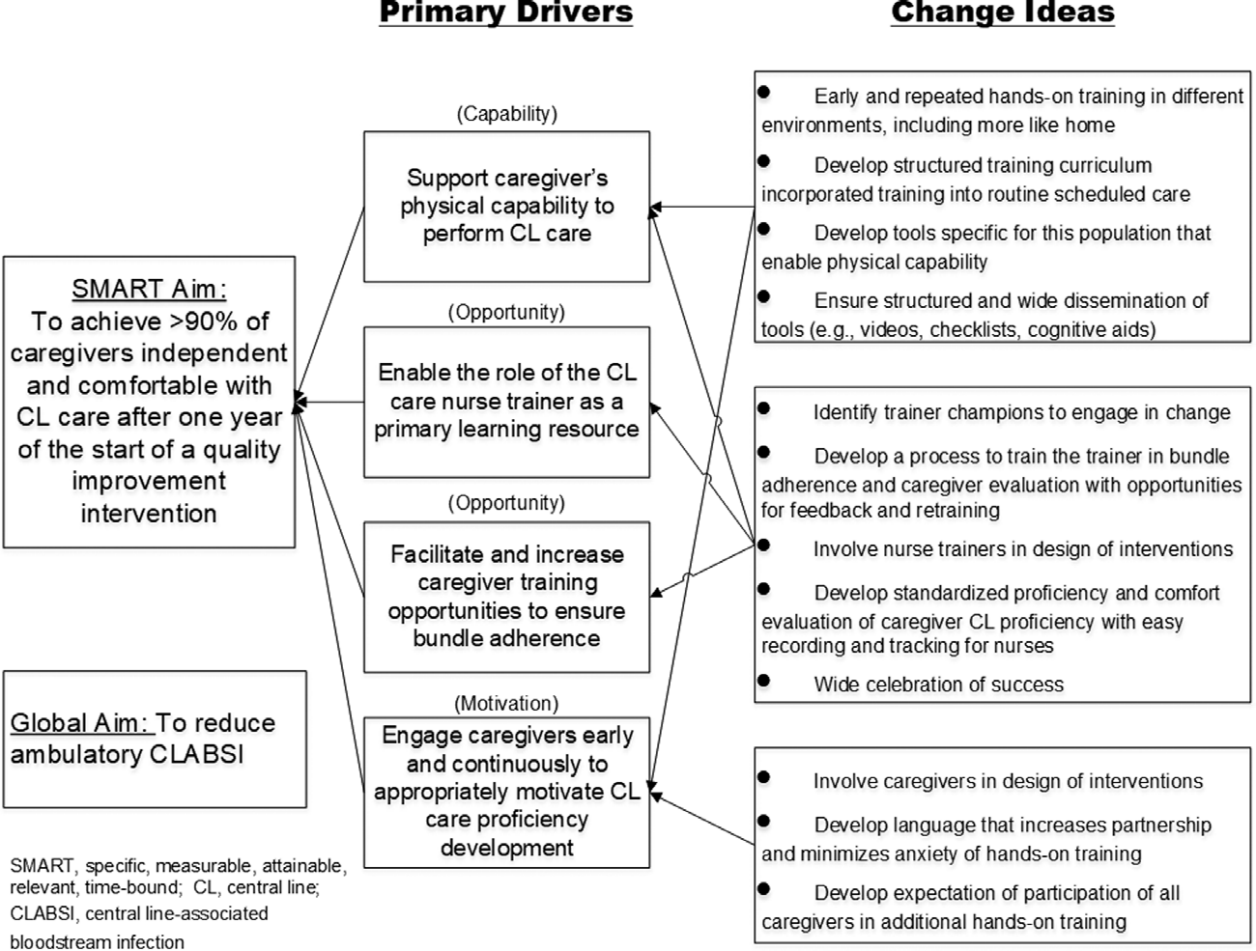
KDD depicting primary drivers and change ideas to optimize caregiver’s independence and comfort with central line care in the home, embedded into capability, opportunity, motivation behavioral model of change.

We identified 4 main drivers of caregiver CL proficiency related to the COM-B model: support the caregiver’s physical capability to perform CL care (capability); enable the role of the CL care nurse trainers as a primary learning resource (opportunity); facilitate and increase caregiver training opportunities to ensure bundle adherence (opportunity); engage caregivers early and continuously to motivate CL care proficiency development (motivation) appropriately. In addition, there were 12 associated change ideas (Fig. 2).

## DISCUSSION

Designing a QI intervention requires understanding the current state and primary drivers that enable change to maximize success. Therefore, we sought caregiver input through a survey and teach-back participation as a CSA to identify the main drivers of caregiver proficiency with CL care, mapped onto the COM-B model, before implementing an intervention.

## CAPABILITY

The psychological aspect of the capability component did not seem to be a main driver of caregiver proficiency, as most answered knowledge questions correctly. At our center, we routinely cover these simple aspects during education; therefore, we expect caregivers to answer most correctly. The physical capability assessment was limited to a few caregivers’ CL care evaluations. Although all were considered independent in CL care, evaluations were not standardized. Most caregivers expressed interest in additional training suggesting skill development needs. Therefore, we proposed supporting the physical aspect of capability as a driver of caregiver CL proficiency. Standardizing caregiver proficiency evaluation would allow for better characterization of this component.

## OPPORTUNITY

From the survey, we gathered that caregivers have some appropriate CL care training resources. The hands-on training was the most valued strategy, indispensable to most. Similar to those of Rinke et al,^1^ our findings support hands-on practice with an expert trainer as key to caregiver’s skill development. During the pilot, nurses did not have time to complete teach-backs, and caregivers suggested standardizing the information received as it was sometimes conflicting. Therefore, we identified enabling the role of the CL care nurse trainer as a primary learning resource key driver. More than 75% of caregivers wanted additional hands-on training opportunities. Despite this interest, we encountered barriers to completing pilot teach-backs without a structured process. This result suggests increasing hands-on training opportunities as a main driver with a proposed intervention of incorporating teach-backs into routine care.

## MOTIVATION

Caregivers had appropriate beliefs about CL care (eg, understood the importance of handwashing). Yet, we did not expect their emotions. Less than half of caregivers had maximal comfort with CL care even after several months. Although we may desire maximal comfort, almost 90% felt some degree of comfort. We did not explore associations that could explain a caregiver’s comfort level. It is difficult to extrapolate responses to 1 survey question to true comfort, especially in an experienced population. Increasing comfort could motivate proficiency development, but we must test this hypothesis. Caregivers’ anxiety with teach-backs as a test suggests the need to develop a language and an approach with caregivers that minimizes this. We did not assess whether caregivers wanted to be proficient in CL care or participate in teach-backs due to social desirability. Therefore, we identified as a driver needing to engage caregivers early and continuously to motivate CL care proficiency development appropriately.

The innovation of this work lies in the intent and approach. We describe our design of a QI intervention by using caregiver input through a CSA to develop key drivers of change. The COM-B model literature mostly identifies barriers/facilitators to behavior change.^30–32^ We utilized the COM-B model to inform an intervention co-design. We chose to focus on addressing care in the home, a unique, uncommon focus of healthcare QI. Rarely is the target of QI a caregiver in the home performing tasks usually done by a trained healthcare worker. Few studies have addressed caregiver CL care learning to reduce ambulatory CLABSI.^1^ Different than Rinke et al, we explored not only the psychological but the physical aspect of the capability component.^1^ We demonstrate that focusing on the physical capability would have a higher value than providing more information (psychological aspect), as most caregivers answered knowledge questions correctly and expressed interest in teach-backs. In addition, we explored the acceptability and challenges of introducing a teach-back as a new training strategy in our center. To our knowledge, reports to reduce ambulatory CLABSI have not used caregiver input to design the intervention or focused mainly on them.^2^ We applied the model of learning from the “frontline worker” (caregiver) to design our intervention. We sought and incorporated caregiver input to co-design the intervention to improve patient outcomes (ambulatory CLABSI rates). Caregiver input provided valuable information for the QI intervention design. For example, we did not anticipate that caregivers would view the teach-back as a test causing anxiety. This finding provides evidence of the importance of co-designing interventions with patients/caregivers, especially when they are key stakeholders.

To achieve our goal of >90% of caregivers independent and comfortable with CL care, our findings suggest the role of a consistent, standardized hands-on training process embedded into routine care to benefit the caregiver. We plan to test a standardized curriculum beginning upon hospital admission. Rather than training caregivers closer to discharge, the early curriculum would allow ample and repeated practice opportunities. Training would be consolidated by an ambulatory teach-back program embedded into routine visits until caregivers are proficient.

The findings have several limitations, reflecting a single institution’s experience with a heterogeneous population, many with significant CL care experience. No validated tools exist to assess caregiver CL care; thus, we mapped data through the COM-B model and relied on assumptions. We evaluated each COM-B model component on a small number of observations or an unstandardized approach. As a result, our data could be subject to selection and social desirability bias. We did not explore contributing factors of comfort level or its association with CL care proficiency. We did not include patients who perform their own CL care, as there are no guidelines or an age cut-off to determine the capacity to do so. Understanding how to support young patients in performing their care is critical. Lastly, we have yet to understand whether addressing these drivers would achieve desired aims.

## CONCLUDING SUMMARY

Engaging key stakeholders and co-designing initiatives is imperative to understanding improvement opportunities and drivers of behavior change. In a priority-payoff matrix, increasing caregiver hands-on opportunities via a standardized process to learn CL care has high impact potential.

## ACKNOWLEDGMENTS

We want to thank Catriona Wagner, who provided medical writing assistance, and Kelly Eng, project manager. Our work would not have been possible without their assistance, and we are very grateful for their contributions. This work was made possible through funding from the Boston Children’s Hospital Program for Patient Safety and Quality Grant, Boston Children’s Hospital Provider and Payor Quality Initiative, and the Cathedral Fund through Ariadne Labs. The authors have no conflict of interest to disclose. Presentation: We presented survey results as part of a selected oral abstract at the 2018 National Patient Safety Foundation Congress.

## Supplementary Material


