# Supplementary material for: Developing the Key Driver Diagram by Analyzing Home Central Line Caregiver Proficiency Factors
Source: Pediatr Qual Saf. 2023 Mar 13;8(2):e638. doi: 10.1097/pq9.0000000000000638 (PMC10013623; doi:10.1097/pq9.0000000000000638)
Supplement: Supplementary file 1 [file pqs-8-e638-s001.pdf]

Logic Model: Ambulatory Central Line-Associated Bloodstream Infection (CLABSI) Prevention Program

Situation: Insufficient training of caregivers performing external central line care is associated with high incidence of preventable ambulatory CLABSI.

Target population: All pediatric hematology, oncology and stem cell transplant ambulatory patients with external central lines.

Goals: 1) Reduce rate of ambulatory CLABSI by 25% relative to baseline within 5 years.
2) Greater than 90% of caregivers comfortable and independent in performing CL care within 1 year.

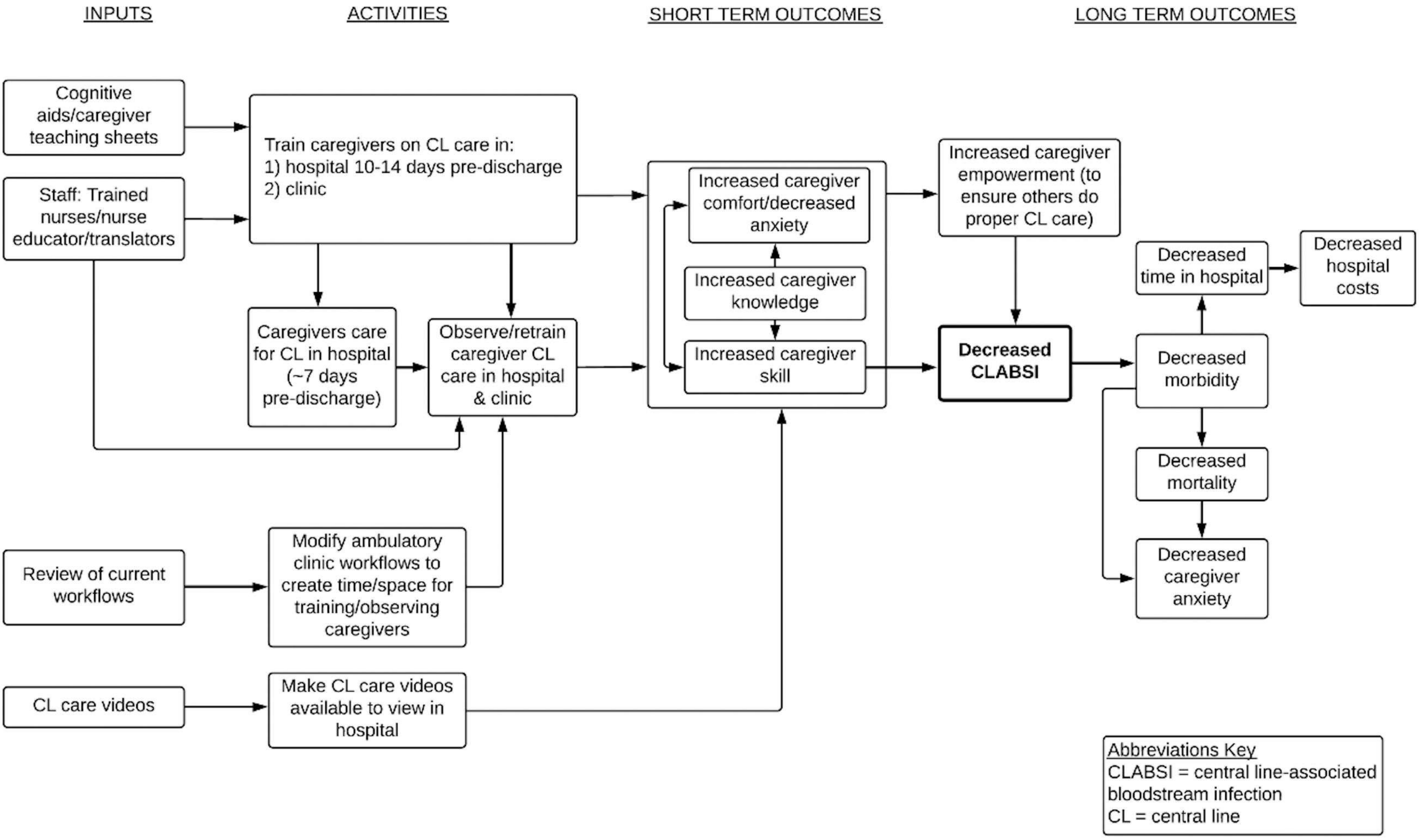

Abbreviations Key
CLABSI = central line-associated bloodstream infection
CL = central line
